# Functional Characterization of the Gibberellin (GA) Receptor ScGID1 in Sugarcane

**DOI:** 10.3390/ijms251910688

**Published:** 2024-10-04

**Authors:** Zhiyuan Wang, Shujun Zhang, Baoshan Chen, Xiongbiao Xu

**Affiliations:** 1 Guangxi Key Laboratory of Sugarcane Biology, College of Agriculture, Guangxi University, Nanning 530004, China; w1274105733@163.com (Z.W.); gxushujunz@126.com (S.Z.); 2 State Key Laboratory for Conservation and Utilization of Subtropical Agro-Bioresources, Guangxi University, Nanning 530004, China; 3 Province and Ministry Co-Sponsored Collaborative Innovation Center of Canesugar Industry, Guangxi University, Nanning 530004, China

**Keywords:** sugarcane, gibberellin, GID1, functional analysis, interaction, regulatory factors

## Abstract

Sugarcane smut caused by *Sporisorium scitamineum* represents the most destructive disease in the sugarcane industry, causing host hormone disruption and producing a black whip-like sorus in the apex of the stalk. In this study, the gibberellin metabolic pathway was found to respond to *S. scitamineum* infection, and the contents of bioactive gibberellins were significantly reduced in the leaves of diseased plants. The gibberellin receptor gene *ScGID1* was identified and significantly downregulated. ScGID1 localized in both the nucleus and cytoplasm and had the highest expression level in the leaves. Eight proteins that interact with ScGID1 were screened out using a yeast two-hybrid assay. Novel DELLA proteins named ScGAI1a and ScGA20ox2, key enzymes in GA biosynthesis, were both found to interact with ScGID1 in a gibberellin-independent manner. Transcription factor trapping with a yeast one-hybrid system identified 50 proteins that interacted with the promoter of *ScGID1*, among which ScS1FA and ScPLATZ inhibited *ScGID1* transcription, while ScGDSL promoted transcription. Overexpression of *ScGID1* in transgenic *Nicotiana benthamiana* plants could increase plant height and promote flowering. These results not only contribute to improving our understanding of the metabolic regulatory network of sugarcane gibberellin but also expand our knowledge of the interaction between sugarcane and pathogens.

## 1. Introduction

*Saccharum* spp., commonly known as sugarcane, is the world’s largest economic sugar crop, with a global production of approximately 1.9 billion tons, and has been cultivated worldwide for centuries [1]. Sugarcane smut, caused by *Sporisorium scitamineum*, is one of the most devastating sugarcane diseases worldwide, causing serious cane yield loss and sugar content reduction [2,3]. The hallmark symptom of sugarcane smut is the development of a black, whip-like sorus at the tip of the infected sugarcane stalk [4,5]. However, early flowering symptoms have been reported upon *S. scitamineum* infection in a certain sugarcane cultivar [6], indicating a specific interaction between the pathogen and host flowering-related genes.

Gibberellin (GA) is a critical phytohormone that plays an important role in various aspects of growth and development [7,8], such as seed germination [9,10,11,12], photomorphogenesis [13,14], flowering regulation [15,16], stamen fertility [17,18] and fruit-setting [19,20]. It is also instrumental in the response to various abiotic stresses [21,22,23,24] and biotic stresses caused by pathogens [25,26,27].

The central GA signaling pathway mainly consists of the GA receptor GIBBERELLIN INSENSITIVE DWARF1 (GID1), DELLA growth inhibitors (DELLAs), F-box proteins, and target genes regulated by DELLA [28,29,30]. Previous work has shown that GA stimulates GA responses via the destruction of DELLA (Asp–Glu-Leu-Leu-Ala) domain proteins, negative regulators of GA responses, via the ubiquitin–proteasome pathway [31]. In general, GA is recognized by the GID1 receptor and assembled into a GID1-GA-DELLA complex; the DELLA is then polyubiquitinated by the Skp1-Cullin-F-box (SCF^SLY1^) E3 ubiquitin–ligase and degraded by the 26S proteasome, and this process is GA dependent [32,33]. Furthermore, a GA-independent DELLA hydrolysis pathway has also been discovered in *Oryza sativa*, *Glycine max*, and *Brassica napus* [34]. It is currently unclear which pathway will be prioritized in integrating external environmental signals or physiological responses and what kind of connection exists between these two pathways.

GID1 is a key mediator in the gibberellin signaling pathway and also the first step in the perception of GA signals by plant cells, with it widely involved in the regulation of the various physiological and biochemical processes of the plant. To date, only a single GID protein has been reported in sugarcane (ScGID1), which can interact with both ScGAI and ScGAIL (a DELLA-like protein lacking the N-terminal domain) and is involved in sugarcane GA signal transduction [35], and it is positively correlated with stalk elongation [36]. However, the interaction and regulatory network and specific mechanism of ScGID1 action remain ambiguous. In our previous studies, *S. scitamineum* infection before black whip sprouting resulted in significantly differential expression of the *ScGID1* gene, suggesting that the GA signaling pathway responds to the infection of *S. scitamineum*.

In this study, a novel *ScGID1* gene named *ScGID1a* was cloned and its tissue expression and subcellular localization were analyzed. A yeast two-hybrid((Y2H) assay was performed to screen and validate proteins that interact with ScGID1. The *cis*-acting regulatory element of the promoter was predicted and the potential core promoter sequence of 500 bp was cloned and verified using a β-Glucuronidase (GUS) histochemical staining assay. The yeast one-hybrid (Y1H) assay was performed to identify the upstream regulatory factors of *ScGID1*, and we found that ScS1FA, ScGDSL, and ScPLATZ can directly interact with the promoter of *ScGID1* and regulate ScGID1 expression. These results enrich the functional study of ScGID1 and shed light on the study of sugarcane gibberellin signaling transduction and flowering regulation.

## 2. Results

### 2.1. The Sugarcane Gibberellin Signaling Pathway Responded to Sporisorium scitamineum Infection

**Figure 1:**
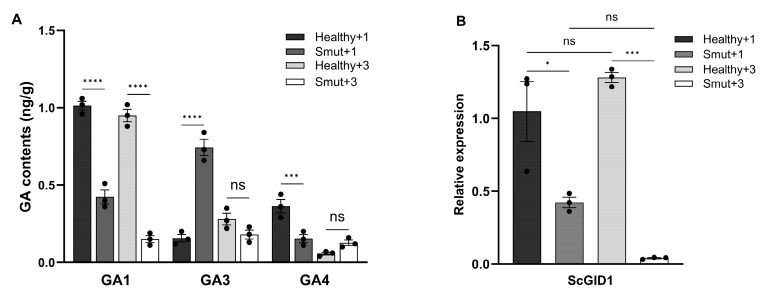
Gibberellin signaling pathway responses to sugarcane smut infection. (**A**) Contents of bioactive gibberellins in smut and healthy sugarcanes. Contents of bioactive gibberellins GA1, GA3 and GA4 in the +1 and +3 leaves of healthy and smut samples were detected by using ESI-HPLC-MS/MS. (**B**) Relative expression level of *ScGID1* in sugarcane leaf tissues during qRT-PCR analysis. The *ScGAPDH* was used as the reference gene. Relative expression levels were calculated with the 2^−ΔΔCt^ method using the mean ± SEM. (The error line indicates the error for three biological replicates. ns, no significant difference. *: *p* < 0.05, ***: *p* < 0.001, and ****: *p* < 0.0001).

To investigate whether black whip formation is involved in the disruption of gibberellin signaling, the contents of bioactive gibberellins of both +1 leaf and +3 leaf of the sugarcanes infected by *S. scitamineum* (but the black whips had not yet germinated) and the healthy sugarcanes in the same growth period were analyzed using ESI-HPLC-MS/MS. As shown in Figure 1, the contents of GA1, GA3, and GA4 in Smut +1 leaves were dramatically reduced when compared to the Healthy +1 leaves (Figure 1A). While in the +3 leaves, the GA1 content in the Smut +3 leaves was significantly lower than that of the healthy ones, there was no significant difference in GA3 and GA4 content between the smut and healthy samples (Figure 1A). The relative expression level of the GA receptor gene *ScGID1* was also analyzed in both smut and healthy leaves using qRT-PCR, and we found that *ScGID1* was dramatically downregulated in both +1 and +3 leaves when infected by *S. scitamineum* (Figure 1B). These results suggest that the host gibberellins signaling pathway is involved in the response to *S. scitamineum* infection, which is consistent with the results reported previously [37], and the *ScGID1* gene plays a significant role in this process.

### 2.2. Identification of the ScGID1 Gene

**Figure 2:**
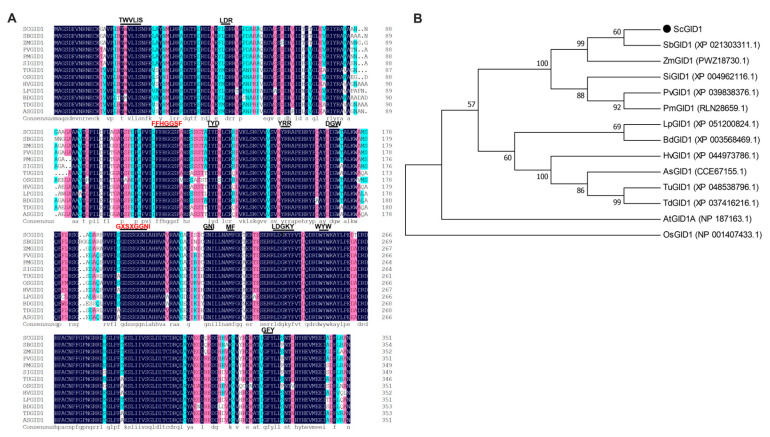
Phylogenetic and expression analysis of ScGID1. (**A**) Multiple sequence alignment between the ScGID1 amino acid sequence and homologues in other species. (TWVLIS, LDR, FFHGGSF, HS, IYD, YRR, DGW, GDSSGGNI, GNI, MF, LDGKYF, WYW, and GFY represent the conserved motifs of GA receptor proteins, marked with black short lines and different fonts. HGG and GXSXG are conserved motifs of the HSL family). (**B**) Phylogenetic analysis of ScGID1 and GID1 homologues in other species. The phylogenetic tree was constructed via the neighbor-joining method by using MEGA11.0 software with the bootstrap value of 1000 replicates. Sb: *Sorghum bicolor*, Zm: *Zea mays*, Si: *Setaria italica*, Pm: *Panicum miliaceum*, Pv: *Panicum virgatum*, Os: *Oryza sativa*, Lp: *Lolium perenne*, Hv: *Hordeum vulgare*, As: *Aegilops speltoides*, Bd: *Brachypodium distachyon*, Td: *Triticum dicoccoides*, Tu: *Triticum urartu*, At: *Arabidopsis thaliana*.

To further explore the molecular function of ScGID1, the coding sequence (CDS) of candidate gene *ScGID1* of 1059 bp was amplified and verified via Sanger sequencing, which encodes 352 amino acids (Figure 2A). When compared with the GIDs from other plants during multi-sequence alignment, we found that the potential ScGID1 obtained here contains all of the conserved motifs of a canonical GA receptor such as TWVLIS, LDR, FFHGGSF, HS, IYD, YRR, DGW, GDSSGGNI, GNI, MF, LDGKYF, WYW, and GFY, as well as the conserved motifs of the hormone-sensitive lipase (HSL) family, including HGG and GXSXG (Figure 2B). In order to further clarify the functional and evolutionary relationships of ScGID1, a phylogenetic tree between ScGID1 and 13 homologues of other plant species (including SbGID1, ZmGID1, SiGID1, PmGID1, PvGID1, etc.) was constructed with MEGA11.0 software using the neighbor-joining method. ScGID1 was closely related to SbGID1 and ZmGID1, and these three proteins were clustered into the same group (Figure 2B), which suggests that ScGID1 is functionally more similar to that in *Sorghum bicolor* and *Zea mays*.

### 2.3. Tissue Expression and Subcellular Location Analysis of ScGID1

**Figure 3:**
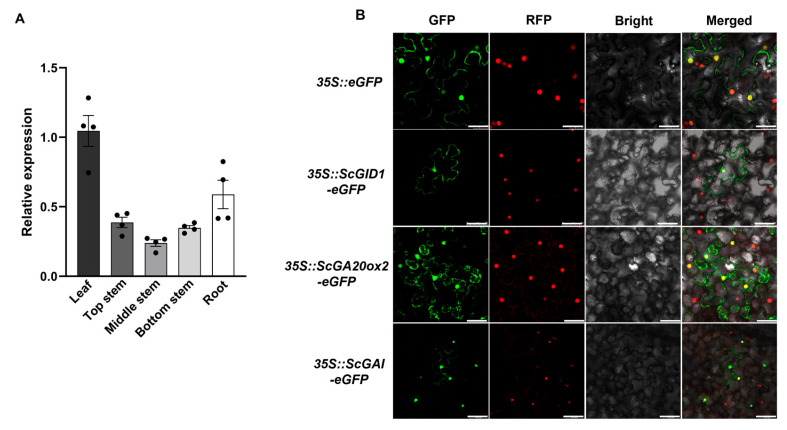
Tissue expression and subcellular location of ScGID1. (**A**) Expression patterns of the *ScGID1* gene in different tissues. Fold changes in the relative gene expression level of all of the other tissues are normalized to the leaf. Three experimental and biological repeats were conducted. Relative expression levels were calculated with the 2^−ΔΔCt^ method using the mean ± SEM (the error line indicates the error for four biological replicates). (**B**) Subcellular localization of ScGID1, ScGA20ox2, and ScGAI in the leaf epidermal cells of RFP-H2B transgenic *N. benthamiana*. GFP, green fluorescent protein; RFP, red fluorescent protein. *35S*::*eGFP* was used as a control; the nucleus-localized RFP−H2B transgenic *N. benthamiana* plants were used as a nuclear marker. This experiment was replicated three times and representative images are displayed. Scale bar = 25 μm.

To determine the expression profiles of *ScGID1* in different tissues, samples from the leaves and the top, middle, and bottom of stems and roots were collected and detected using qRT−PCR. As shown in Figure 3A, the expression profiles of *ScGID1* exhibit obvious tissue specificity, with the highest expression levels in the leaves and the lowest expression levels in the middle stems (Figure 3A). Furthermore, an *eGFP* fluorescent reporter gene was fused to the C-terminus of ScGID1 (*35S*::*ScGID1*-*eGFP*), and the subcellular localization of ScGID1 was investigated in RFP-H2B transgenic *Nicotiana benthamiana* via transient expression. Strong green fluorescence was observed in the nucleus and cytoplasm of transgenic *N. benthamiana* mesophyll cells infiltrated with *35S*::*ScGID1*-*eGFP*. The control recombinant of *35S::eGFP* also showed similar subcellular localization (Figure 3B), indicating that the ScGID1 protein is localized in the nucleus and cytoplasm when transiently expressed in *N. benthamiana*, which is consistent with the results reported in sugarcane [35].

### 2.4. ScGID1 Interacts with Key Proteins in Gibberellin Biosynthesis and Metabolism Pathways

**Figure 4:**
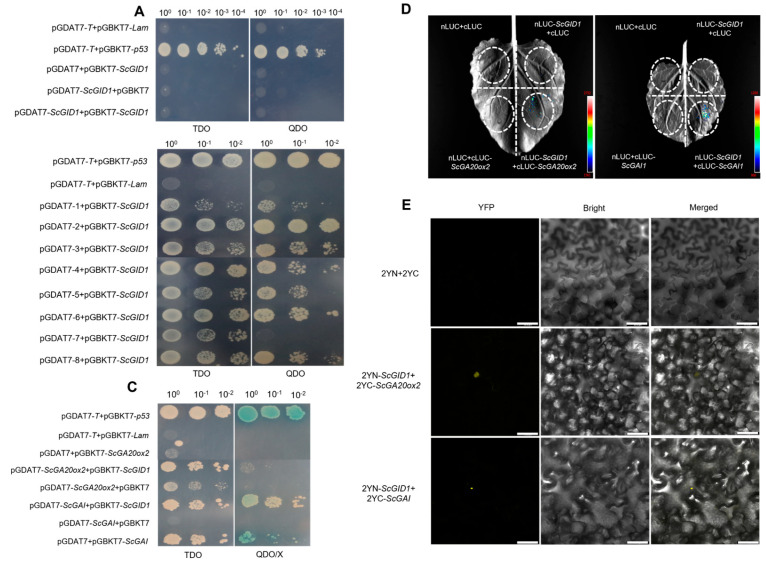
Interaction analysis between ScGID1 and host factors. (**A**) Autoactivation of pGBKT7-*ScGID1* in yeast Y2HGold. (**B**) Interactions between ScGID1 and eight proteins in sugarcane via the yeast two-hybrid assay. (**C**) Yeast two-hybrid assays showing the interactions between ScGID1 and ScGAI or ScGA20ox2. pGBKT7-*p53*+pGADT7-*T* was used as the positive control, and pGBKT7-*Lam*+pGADT7-*T* was used as the negative control. TDO: SD/−Leu/−Trp/−His triple dropout, QDO: SD/−Leu/−Trp/−His/−Ade quadruple dropout, QDO/X: SD/−Leu/−Trp/−His/−Ade quadruple dropout + X-α-gal. (**D**) Verifying the interaction between ScGID1 and ScGA20ox2 or ScGAI by splitting luciferase assays in *N. benthamiana* leaves. nLUC + cLUC, nLUC-*ScGID1* + cLUC, nLUC + cLUC-*ScGA20ox2*, and nLUC + cLUC-*ScGAI* were used as the negative controls. (**E**) BiFC analyses of ScGID1 and ScGA20ox2/ScGAI interactions in *N. benthamiana* leaves. 2YN+2YC was used as the negative control. Scale bar = 36 μm. All of the experiments were replicated three times.

To investigate the potential interaction network of ScGID1, a sugarcane cDNA library was constructed and screened via a yeast two-hybrid (Y2H) by using pGBKT7-*ScGID1* as the bait, and it was proven to show no toxicity and self-activation activity (Figure 4A). After high-strength screening on SD/−Trp/−Leu/−His (TDO) and SD/−Trp/−Leu/−His/−Ade (QDO) media, the potential positive clones were selected and sequenced. After the exclusion of repeats, the remaining eight clones were retained and annotated in the Sugarcane Genome Hub database (https://sugarcane-genome.cirad.fr access date: 12 November 2023) (Table 1), and point-to-point interaction validation was performed (Figure 4B). Taken together, these annotations suggest that ScGID1 can interact with various proteins related to plant secondary metabolite synthesis, growth metabolism, hormone response, and stress tolerance.

To date, ScGID1 is the only gibberellin receptor found in sugarcane, and little is known about its biological function. To investigate the possible role of ScGID1 and the interaction between ScGID1 and key factors in gibberellin synthesis and metabolism, a yeast two-hybrid assay was performed. The CDSs of *ScGA20ox2* and *ScGAI* were amplified and cloned into the pGADT7 vector, respectively. The recombinant prey plasmids were co-transformed with pGBKT7-*ScGID1* into Y2HGold yeast cells. The results showed that both ScGA20ox2 and ScGAI can interact with ScGID1 in the absence of GA3, and the interaction between ScGA20ox2 and ScGID1 is much weaker than that of ScGAI-ScGID1 (Figure 4C). The split-luciferase assays also confirmed similar results (Figure 4D). BiFC assays were also performed to confirm the interactions between ScGID1 and ScGA20ox2 or ScGAI, as shown in Figure 4E. ScGAI can interact with ScGID1 in the nucleus (Figure 4E), which is consistent with the results reported previously [35]. Also, ScGA20ox2 was found to interact with ScGID1 in the nucleus too.

### 2.5. A Novel GAI1a Homologue in Sugarcane Interacts with ScGID1 and Its Mutants in a Gibberellin-Independent Manner

**Figure 5:**
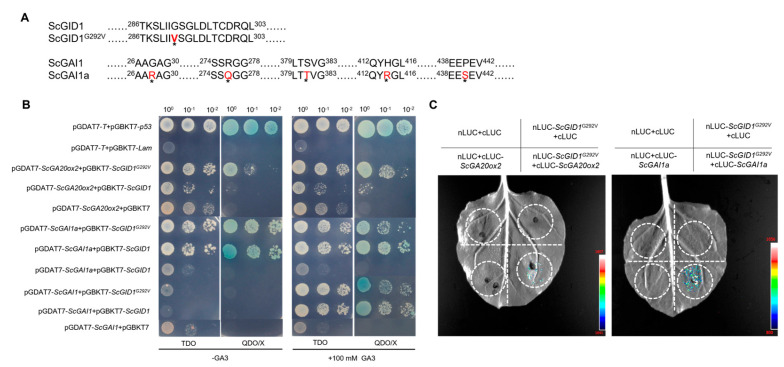
Interactions of wild-type ScGID1 and its mutant with ScGAI1a and ScGA20ox2. (**A**) Schematic diagram of the amino acid sequence of ScGID1, ScGAI, and their homologues. (**B**) Verifying the interaction between ScGID1 and ScGA20ox2 or ScGAI via the yeast two-hybrid assay. pGBKT7-*p53*+PGADT7-*T* was used as the positive control, and pGBKT7-*Lam*+PGADT7-*T* was used as the negative control. TDO: SD/−Leu/−Trp/−His triple dropout, QDO/X: SD/−Leu/−Trp/−-His/−Ade quadruple dropout + X-α-gal. (**C**) Verifying the interaction between ScGID1^G292V^ and ScGA20ox2 or ScGAI1a using the splitting luciferase assays. nLUC + cLUC, nLUC-*ScGID1*^G292V^ + cLUC, nLUC + cLuc-*ScGA20ox2*, and nLUC + cLuc-*ScGAI1a* were used as the negative controls.

Previous reports suggest that the presence of gibberellin is essential for the interaction between ScGID1 and ScGAI [35], which is inconsistent with our results in this study. Since sugarcane is a polyploid plant, there may be multiple alleles on its chromosome, and an amino acid difference at position 292 with a valine mutated to glycine was found between the ScGID1^G292V^ amplified here and that reported previously. And there were 5 amino acid differences between the ScGAI obtained in this study (hereinafter referred to as ScGAI1a) and previous reports (Figure 5A). 

To investigate whether the amino acid difference is the cause of the GA-independent interaction between GID1 and GAI1 and whether ScGID1^G292V^ affects its interaction with ScGA20ox2, the plasmids pGADT7-*ScGA20ox2* and pGADT7-*ScGAI1,* or pGADT7-*ScGAI1a* were constructed and co-transformed with pGBKT7-*ScGID1*^G292V^ or pGBKT7-*ScGID1* into Y2HGlod yeast strains, respectively. The results show that both ScGID1^G292V^ and ScGID1 can interact with ScGA20ox2 in a gibberellin-independent manner. The interaction between ScGAI1 and ScGID1^G292V^ or ScGID1 did depend on gibberellin, which is consistent with the results of Chai and colleagues [35]. To our surprise, ScGAI1a can interact with both ScGID1^G292V^ and ScGID1 in the absence of gibberellin, and the G292V mutation of ScGID1 did not affect its interaction with ScGAI1 and ScGAI1a (Figure 5B). Similar results were also found in the in vivo split-luciferase assay (Figure 5C). The above results indicate that ScGAI1a is a novel DELLA protein that interacts with ScGID1 in a gibberellin-independent manner, and ScGID1 can function as a GA receptor in gibberellin signaling transduction on the one hand and simultaneously regulate upstream GA biosynthesis by interacting with ScGA20ox.

### 2.6. Potential Promoter of ScGID1 and Its Upstream Regulatory Transcription Factors

**Figure 6:**
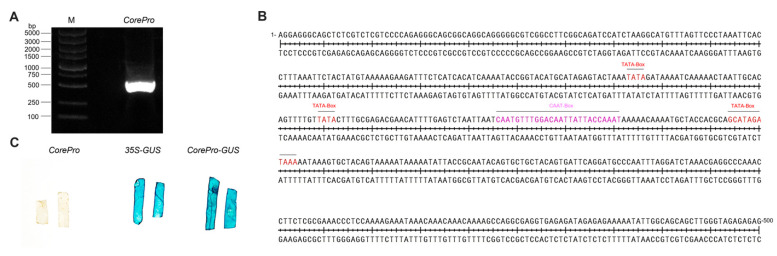
*ScGID1* promoter cloning and promoter activity analysis. (**A**) Amplifying and electrophoresis of the core promoter sequence of *ScGID1*. M: DNA marker, *CorePro*: core promoter sequence of *ScGID1*. (**B**) Core promoter and cis-acting elements prediction of *CorePro* using the PlantCARE database. The core TATA-box and CAAT-box elements were labeled with red and pink, respectively. (**C**) Analysis of the promoter activity of *ScGID1 CorePro* using GUS histochemical staining in *N. benthamiana*. pCHF3-*CorePro* (*CorePro*) and pCHF3-*35S*-*GUS* (*35S-GUS*) were used as the negative control and positive control, respectively.

To identify the potential upstream regulatory transcription factors (TFs) of *ScGID1*, a DNA fragment of 2000 bp upstream of the *ScGID1* gene was analyzed on the PlantCARE online website [38], and the potential *cis*-acting elements were analyzed. In addition to the common *cis*-acting and core promoter elements such as CAAT-box and TATA-box, there are also a series of important conserved elements such as abscisic acid-related AAGAA-motif, photo-responsive G-box, MYB-binding, STRE stress-responsiveness, etc. (Table S2), suggesting its promoter activity. A potential core promoter region between 562 nt and 1061 nt upstream of the *ScGID1* start codon was further amplified, which is enriched with CAAT-box and TATA-box motifs (hereafter referred to as *CorePro*) (Figure 6A). The potential *CorePro* was inserted into the plant binary expression vector pCHF3 to replace the *CaMV 35S* promoter, and the recombinant plasmid was named pCHF3-*Core*. The β-glucuronidase (GUS) reporter gene was inserted into MCS to obtain pCHF3-*CorePro*-*GUS*. The transcriptional activity of *CorePro* was measured using *N. benthamiana* GUS histochemical staining. As we can see in Figure 6C, the *N. benthamiana* leaf chips infiltrated with the pCHF3-*CorePro* empty vector (negative control) remained transparent after ethanol decoloring, and the leaves infiltrated with pCHF3-*CorePro*-*GUS* showed a deeper blue than that of pCHF3-*35S*-*GUS* (positive control) (Figure 6C). The results here suggest that the *CorePro* of *ScGID1* has strong promoter activity, with it stronger than that of *CaMV 35S*.

**Figure 7:**
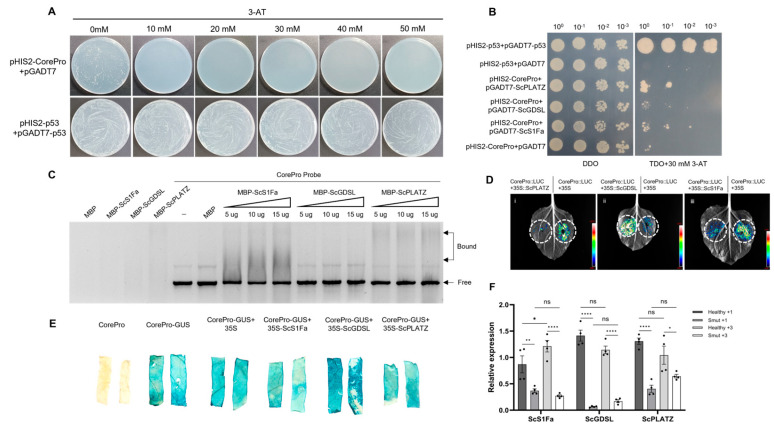
The core promoter of *ScGID1* interacts with ScS1Fa, ScGDSL, and ScPLATZ. (**A**) Self-activation detection of decoy vector yeast single hybridization. (**B**) Validation of the interaction between *ScGID1 CorePro* and ScS1Fa, ScPLATZ, and ScGDSL using the yeast one-hybrid. DDO: SD/−Leu/−Trp double dropout, TDO: SD/−Leu/−Trp/−His triple dropout. (**C**) EMSA analyses of the binding of ScS1Fa, ScPLATZ, or ScGDSL to *CorePro*. (**D**) Luminescence images showing the effects of regulatory factors on *CorePro-LUC* transcription. (i), (ii), and (iii) represent the effect of ScS1Fa, ScGDSL and ScPLATZ on *CorePro* transcription activity, respectively. The pCHF3 empty vector was used as a negative control. (**E**) Analysis of the effects of ScS1Fa, ScGDSL, and ScPLATZ on *ScGID1* promoter activity via GUS histochemical staining. pCHF3-*CorePro* (*CorePro*), pCHF3-*CoreProGUS* (*CorePro*-*GUS*), and pCHF3*CorePro*-*GUS*+pCHF3 (*CoreProGUS*+*35S*) were used as negative controls. (**F**) Relative expression levels of *ScS1Fa*, *ScGDSL,* and *ScPLATZ* in sugarcane infected with *Sporisorium scitamineum*. *ScGAPDH* was used as a reference gene, and relative expression levels were calculated with the 2^−ΔΔCt^ method using mean ± SEM. (The error line indicates the error for four biological replicates. ns: no significant difference. *: *p* < 0.05, **: *p* < 0.01, and ****: *p* < 0.0001). All of the experiments were repeated three times, and a representative image is shown.

To investigate the potential upstream regulatory factors of *ScGID1*, a Y1H assay was performed. We found that a concentration of 30 mM 3-AT effectively inhibited the self-activation activity of the pHIS2-*CorePro* reporter plasmid (Figure 7A). The Y187 yeast cells transformed with pHIS2-*CorePro* were used as recipient strains for sugarcane cDNA library plasmid transformation. The yeast transformants were cultured on TDO plates containing 30 mM 3-AT. A total of 288 positive yeast clones were screened, all of which were amplified by PCR and sequenced. After excluding repeats, 50 different sequences were finally obtained after BLAST comparison (Table S3). The above 50 positive clones were verified through co-transformation with the pHIS2-*CorePro* bait plasmid, and all of these potential positive clones could grow on DDO, TDO, and QDO + 30 mM 3-AT-deficient media (Figure S1).

ScGID1, as a gibberellin receptor, is closely related to host growth and development. Genes involved in plant growth and development, such as ScS1Fa, ScGDSL, and ScPLATZ, were cloned based on the Sugarcane Genome Hub database. In order to verify whether these proteins can bind to the *CorePro* of *ScGID1*, the complete coding sequences of ScS1Fa, ScGDSL, and ScPLATZ were amplified and inserted into the pGADT7 vector. Y1H assays showed that ScS1Fa, ScPLATZ, and ScGDSL can bind to the *CorePro* region of the ScGID1 promoter (Figure 7B). Similar results were also found in the EMSA assays which showed that ScS1Fa and ScPLATZ could bind to the *CorePro* of *ScGID1*, while no detectable binding activity was observed between ScGDSL and *CorePro* (Figure 7C), which indicates that ScGDSL may interact with *ScGID1* promoter via some bridge proteins. Furthermore, dual-luciferase activity assays were performed, and the leaf areas infiltrated with ScPLAZT and ScS1Fa showed much weaker fluorescence than those infiltrated with the *35S* promoter, while leaves infiltrated with ScGDSL showed much stronger fluorescence than those of the 35S promoter control (Figure 7D). GUS histochemical staining assays showed that the leaves co-infiltrated with *CorePro*-*GUS* and ScGDSL exhibited much deeper blue precipitates than those infiltrated with *CorePro-GUS* or *CorePro*-*GUS*+*35S*, while the leaf chips co-infiltrated with *CorePro*-*GUS*+*ScS1Fa* and *CorePro*-*GUS*+*ScPLATZ* showed lighter blue precipitates when compared with the *CorePro*-*GUS* or *CorePro*-*GUS*+*35S* controls (Figure 7E). These results indicate that both ScPLAZT and ScS1Fa repress the transcription of *ScGID1*, while ScGDSL promotes its transcription.

The relative expression levels of *ScS1Fa*, *ScGDSL,* and *ScPLATZ* were also analyzed when *S. scitamineum* infected the sugarcane plants. As we can see in Figure 7F, all three genes were significantly downregulated in both +1 and +3 leaves in the smut sugarcane samples when compared with those in the healthy sugarcane (Figure 7F), indicating that ScS1Fa, ScGDSL and ScPLATZ are involved in *S. scitamineum* infection and regulation of the induced gibberellin metabolism.

### 2.7. Overexpression of ScGID1 Promotes the Growth of N. benthamiana

To further investigate the biological function of ScGID1 in sugarcane, *35S*::*3×Flag*-*ScGID1* and *35S*::*3×Flag* recombinant overexpression plasmids were constructed and transformed into *N. benthamiana* through *Agrobacterium*-mediated leaf disc transformation. Eight of both *3×Flag*-*ScGID1* and *3×Flag* transgenic overexpression lines were obtained, and the positive overexpression seedlings were validated via PCR and qRT-PCR analysis (Figure S2). The representative *ScGID1*-overexpressing transgenic lines were then transplanted into the field for further phenotypic observation.

**Figure 8:**
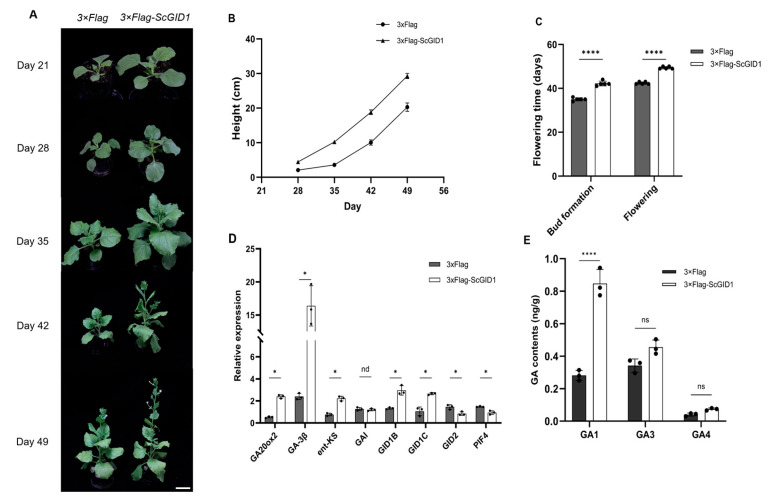
Phenotypes of *ScGID1*-overexpressing transgenic plants and relative expression levels of genes involved in GA signal biosynthesis and metabolism in *N. benthamiana*. (**A**) Growth and development processes of *3×Flag*-*ScGID1* and *3×Flag* transgenic *N. benthamiana* plants. The photographs were taken at 21, 28, 35, 42, and 49 days after transplantation, respectively. Scale bar = 3 cm. (**B**) The height of the *3×Flag*-*ScGID1* transgenic and control plants in different periods. (**C**) Flowering time of the *3×Flag*-*ScGID1* transgenic and control plants. Five individual plants of both *3×Flag*-*ScGID1* and *3×Flag* transgenic plants were analyzed. (**D**) Relative expression levels of genes involved in GA signal biosynthesis and metabolism in *N. benthamiana*. *NbActin* was used as a reference gene. Three individual plants of *3×Flag*-*ScGID1* and *3×Flag* transgenic plants were analyzed. (**E**) Contents of bioactive gibberellins in *3×Flag*-*ScGID1* and *3×Flag* transgenic plants. ns: no significant difference. *: *p* < 0.05, and ****: *p* < 0.0001.

The T1 generation seedlings capable of stably expressing *3×Flag*-*ScGID1* or *3×Flag* were cultured in an insect-free chamber and observed for a consecutive period of 7 weeks. As we can see in Figure 8A, the blade area of the *3×Flag*-*ScGID1* transgenic plants was larger than that of the control (Figure 8A). The seedling height of the *3×Flag*-*ScGID1* or *3×Flag* transgenic plants was measured every 7 days, and we found that the height of the *3×Flag*-*ScGID1* transgenic seedlings was significantly higher than that of the control plants of the same age (Figure 8B). Moreover, most of the *3×Flag*-*ScGID1* transgenic plants showed flower buds on the 35th day after transplantation, while the *3×Flag* control plants did not show any flower buds. In addition, 42 days after transplantation, almost all of the *3×Flag*-*ScGID1* transgenic plants were in full bloom, while the control plants were still in the flower bud development stage, and 49 days after planting, the control plants began to bloom. Subsequently, the flower buds of the *3×Flag*-*ScGID1* transgenic plants appeared and flowered approximately 7 days earlier than those of the control plants (Figure 8C). These results suggest that *ScGID1* can promote early flowering and plant height in *N. benthamiana*.

To further reveal how ScGID1 influences host growth and flowering regulation, genes involved in gibberellin synthesis (*ent*-*KS*, *GA-3β,* and *GA20ox*), signal transduction (*GID1B* and *GID1C*), and regulatory pathways (*GAI*, *GID2,* and *PIF4*) were analyzed via qRT-PCR in *3×Flag*-*ScGID1* and *3×Flag* transgenic *N. benthamiana* plants 35 days after germination. As shown in Figure 8D, the relative expression levels of *ent*-*KS* (*ent*-kaurene synthase), *GA-3β* (GA 3beta-hydroxylase), and *GA20ox* genes involved in gibberellin synthesis were upregulated in *3×Flag*-*ScGID1* transgenes when compared with the *3×Flag* control, and two gibberellin receptor genes, *GID1B* and *GID1C,* were also upregulated and the gibberellin negative regulators *GID2* and *PIF4* were significantly downregulated, while there was no significant difference in *GAI* gene expression (Figure 8D). The contents of bioactive gibberellins were also analyzed via ESI-HPLC-MS/MS, and the results showed that the contents of GA1, GA3, and GA4 in *3×Flag*-*ScGID1* transgenic plants were significantly higher than that of the control plants, the contents of GA1, GA3, and GA4 were 3-fold, 1.3-fold, and 1.8-fold higher than those of the control plants, respectively (Figure 8E). The results of the qRT-PCR assay are consistent with the detection of hormone levels in transgenic plants, suggesting that constitutive overexpression of *ScGID1* can promote the growth of *N. benthamiana* plants by regulating gibberellin biosynthesis and metabolism.

## 3. Discussion

Sugarcane smut caused by *Sporisorium scitamineum* is a destructive disease throughout the world, and a lot of metabolic pathways and biological processes have been demonstrated to be associated with *S. scitamineum* infection, such as differentially expressed genes involved in pathogen resistance, flavonoid and phytohormone synthesis, etc. [37,39]. In our previous work, a gibberellin receptor ScGID1 was found to be dramatically repressed in *S. scitamineum-infected* leaves by RNA-Seq. Furthermore, the contents of bioactive gibberellins GA1, GA3, and GA4 were significantly different between *S. scitamineum-infected* and healthy plants (Figure 1). This indicates that the host gibberellin signaling pathway is involved in the response to *S. scitamineum* infection and that ScGID1 may play a critical role in this process.

*GID1* is a key point in the GA signaling pathway and was first cloned from rice [28]. It has been confirmed that GID1 can interact with GAI and form a GA-GID1-DELLA complex, which plays a significant role in regulating plant height, flower development, and fruit length [40,41]. In this study, the complete CDS of *ScGID1* was cloned from sugarcane, multiple sequence alignment was performed, and we found that ScGID1 belongs to the α/β hydrolase superfamily and possesses all the conserved motifs of GA receptors, including the conserved HGG and GXSXG [33]. Phylogenetic analysis also showed a distinct genetic evolutionary relationship of ScGID1 with the homologues compared and had the closest relationship with SbGID1 (Figure 2). 

In higher plants, gibberellin biosynthesis is a complex, multi-stepped process with various intermediates, including *ent*-Kaurene synthesis in the plastid, the conversion of *ent*-Kaurene to *ent*-Kaurenoic acid on the outer plastid membrane, and the conversion of *ent*-Kaurenoic acid to GA12 and subsequent GA53, GA1, GA4 in the endoplasmic reticulum and cytoplasm, respectively [7,10,42]. Then, the bioactive gibberellins are transported upward or downward to the tissues where they act through several GA influx transporters [43] and are eventually recognized by GA receptor GID1 [28,44,45]. It has been previously reported that GID1 proteins are located mainly in the nucleus, but they are also distributed in the cytoplasm [28,46,47], and in this study, we found that the ScGID1 protein localizes in both the nucleus and cytoplasm (Figure 3), which was consistent with the results of Chai and colleagues and that of EuGID1 in *Eucommia ulmoides* Oliver [35,48]. We found that ScGID1 was expressed in all organs of sugarcane, with the highest level in the leaves, suggesting a synergistic effect of ScGID1 and GA in the development of sugarcane leaf morphology.

The gibberellin metabolic pathway in plants is a complex network involving a number of genes, enzymes, and regulators. In this study, eight candidate host factors that interact with ScGID1 were identified via Y2H. These eight candidate proteins play important roles in plant secondary metabolite synthesis, growth and development, hormone response, and stress resistance, which indicates that ScGID1 may also play an important role in many biological processes in addition to its vital role in gibberellin metabolism. 

GID1 proteins are important factors in GA perception in both dicots and monocots [45]. Usually, GID1 mainly interacts with the downstream DELLA protein in a GA-dependent manner and leads to the degradation of DELLA via the 26S ubiquitin–proteasome pathway [32,33]. However, the GA-independent interaction between GIDs and DELLA has also been confirmed to be an important pathway in GA-signal transduction [34]. Due to its complex polyploid genome, there may be multiple alleles of DELLA in sugarcane, and in this trial, two DELLA proteins, ScGAI1 and ScGAI1a, were cloned. As expected, the results of the Y2H assay show that ScGAI1 can interact with ScGID1 in a GA-dependent manner, which is consistent with the results of Chai and colleagues [35]. To our surprise, we found that ScGAI1a can interact with ScGID1 both in the presence and absence of GA, indicating a gibberellin-dependent manner (Figure 5). Sequence alignment shows a 5-amino acid difference at position G28R, R276Q, S381T, H414R, and P440S between ScGAI1 and ScGAI1a, which suggests that ScGAI1a is a novel DELLA protein and may play an important role in sugarcane gibberellin signal regulation in a GA-independent manner. Meanwhile, we found that ScGID1^G292V^ did not affect its interaction with ScGAI1 and ScGAI1a, while it showed a stronger interaction with ScGA20ox2 than the reported ScGID1, and this type of interaction was gibberellin independent. The interaction between ScGID1 and ScGA20ox2 and the significant role of GA20ox2 in catalyzing the final steps of bioactive gibberellins synthesis [49,50] suggest that ScGID1 participates in host gibberellin metabolism not only by acting as an intrinsic GA receptor but also regulates GA biosynthesis by interacting with ScGA20ox2.

Y1H is a powerful and widely used tool to identify DNA–protein interactions, such as TFs [51]. GID1-GA recognition is considered as the first step in GA signal transduction and then activates downstream DELLA-related degradation and participates in lots of plant hormone metabolic pathways [10,28,52]. To date, little is known about the plant GID1-interacting network and upstream regulators. In this study, 50 candidate TFs upstream of *ScGID1*, including ScS1FA, ScGDSL, and ScPLATZ, were identified via YIH screening and verification (Figure 7 and Figure S1), which helps to shed light on illustrating the role of GID1 in the plant hormone regulatory network.

S1Fa-like TFs are small proteins with both nuclear localization and DNA binding domains and play important roles in abiotic stress and hormonal response [53,54,55]. The S1Fa in spinach was found to be related to photomorphogenesis and its expression level was affected by light [56]. Four S1Fa TFs were found to be upregulated in soybean after treatment with paclobutrazol, an inhibitor of gibberellins, suggesting that S1Fas may function as negative regulators of gibberellin signaling and be involved in photosynthetic growth [57]. As previously reported, the expression of *GID1* is under the control of the circadian clock, meaning that light is involved [58]. In this study, we found that ScS1Fa negatively regulates the expression of *ScGID1* (Figure 7D,E) and was downregulated upon *S. scitamineum* infection (Figure 7F). However, *ScGID1* was significantly downregulated in *S. scitamineum*-infected leaves, as we found previously (Figure 1B), which suggests that other factors apart from ScS1Fa may be involved in *ScGID1* regulation. 

GDSL-lipases represent a class of lipolytic enzymes that play vital roles in plant growth and development, organ morphogenesis, secondary metabolism, plant immunity, and stress response [59,60]. Compared with wild-type plants, the seed germination rate and seedling survival rate of BnGDSL1 overexpressing lines were higher in *Brassica napus* [61]. Loss of ZmMs30 function in *Zea mays* results in other cuticle defects, pollen outer layer irregularities, and complete sterility in males [62]. The transcripts of *CaGLIP1* in *Capsicum annuum* were upregulated in response to a variety of defense hormones such as salicylic acid (SA), jasmonic acid (JA), and ethylene (ET), and CaGLIP1 was proven to be involved in the defense against *Xanthomonas campestris* and *Pseudomonas syringae* [63]. Here, in this work, we found that ScGDSL promotes the expression of *ScGID1* and is downregulated in response to *S. scitamineum* infection (Figure 7D,E), suggesting that ScGDSL may be involved in host gibberellin metabolism by regulating the expression of *ScGID1* on the one hand and defense response to sugarcane smut on the other. 

Plant A/T-rich sequence and zinc-binding proteins (PLATZ) are plant-specific TFs involved in growth, development, and stress responses and are widely distributed in monocots, dicots, mosses, and algae [64,65]. In *Arabidopsis*, ORESARA15 (ORE15) can regulate leaf growth and senescence [66]. The PLATZ-A1 TF in wheat has been reported to interact with a DELLA protein (RHT1) and control plant height [67]. As we report here that ScPLATZ can bind to the promoter sequence of *ScGID1* and repress its transcription (Figure 7C–E), together with the interaction between ScGID1 and ScGAI (Figure 4), we speculate that, on the one hand, ScPLATZ can directly interact with ScGAI and, on the other, control *ScGID1* transcription to regulate GA signal transduction at the same time.

As an important plant hormone, gibberellin also regulates the transition from vegetative to reproductive growth and promotes flowering [68,69]. After overexpression of *ScGID1* in *N. benthamiana*, the transgenic plants were found to be taller than the control and exhibited an obvious early flowering phenotype (Figure 8), which was consistent with previous studies [48,70]. Furthermore, the expression levels of positive regulators of gibberellin synthesis factors, such as *ent*-*KS*, *GA-3β*, and *GA20ox2* were significantly upregulated in *ScGID1* transgenic *N. benthamiana* plants, while the negative gibberellin signaling pathway regulators *GID2* and *PIF4* were downregulated (Figure 8D). The content of bioactive GA1 was much higher in *ScGID1* overexpressing transgenic plants than that of the control (Figure 8E), which is consistent with the phenotype of higher plants. Therefore, overexpression of the *ScGID1* gene had a significant impact on gibberellin biosynthesis and plant growth and development. It is reasonable to speculate that overexpression of the *ScGID1* gibberellin receptor gene helps accelerate plant growth, increase plant height, and promote flowering in plants.

## 4. Materials and Methods

### 4.1. Plant Materials and Treatments

*Nicotiana benthamiana* and RFP-H2B transgenic *N. benthamiana* seedlings were grown in an insect-free growth chamber at 25 °C and 60% relative humidity under a 16 h light/8 h dark photoperiod. The leaf, stem, and roots of a healthy ROC22 sugarcane cultivar were collected in Nanning, Guangxi Zhuang Autonomous Region. Samples were collected and mixed evenly and quickly frozen in liquid nitrogen and stored at −80 °C.

### 4.2. Gene Cloning and Sequence Analysis

RNA was extracted from leaf samples using the RNA isolater Total RNA Extraction Reagent (Cat. No. R401-01, Vazyme, Nanjing, China) according to the manufacturer’s instructions. cDNA was synthesized from RNA using a HiScript III 1st Strand cDNA Synthesis Kit (+gDNA wiper) (Cat. No. R312-02, Vazyme, Nanjing, China). The coding regions of the *ScGID1* (GenBank accession number: QAA78878), *ScGAI* (GenBank accession number: QAA78876), *ScGAI1a* (GenBank accession number: PQ045663), *ScGA20ox2* (GenBank accession number: PQ045660), *ScS1Fa* (GenBank accession number: PQ045659), *ScGDSL* (GenBank accession number: PQ045661), and *ScPLATZ* (GenBank accession number: PQ045662) genes were amplified from the cDNA of the sugarcane cultivar ROC22 using gene-specific primers (Supplementary Table S1). The resulting PCR amplicons were purified and cloned into the pCE2-TA/Blunt-Zero vector (Cat. No.C601-01, Vazyme, Nanjing, China), respectively. The corresponding recombinant plasmids were transformed into *Escherichia coli* DH5α and verified via colony PCR and Sanger sequencing (Sangon Biotech, Shanghai, China). Amino acid sequences of ScGID1 and homologs annotated as GID1 proteins in a variety of other plants were downloaded from GenBank: SbGID1 (*Sorghum bicolor*, XP_021303311.1), ZmGID1 (*Zea mays*, PWZ18730.1), SiGID1 (*Setaria italica*, XP_004962116.1), PmGID1 (*Panicum miliaceum*, RLN28659.1), PvGID1 (*Panicum virgatum*, XP_039838376.1), OsGID1 (*Oryza sativa* Japonica Group, NP_001407433.1), LpGID1 (*Lolium perenne*, XP_051200824.1), HvGID1 (*Hordeum vulgare* subsp. *Vulgare*, XP_044973786.1), AsGID1 (*Aegilops speltoides*, CCE67155.1), BdGID1 (*Brachypodium distachyon*, XP 003568469.1), TdGID1 (*Triticum dicoccoides*, XP_037416216.1), TuGID1 (*Triticum urartu*, XP_048538796.1), and AtGID1A (*Arabidopsis thaliana*, NP_187163.1). Multiple sequence alignment was conducted using DNAMAN. The phylogenetic tree of ScGID1 and homologues was constructed via the neighbor-joining method by using MEGA11.0 software with the bootstrap value of 1000 replicates.

### 4.3. Real-Time Fluorescence Quantitative PCR (RT-qPCR)

Each RT-qPCR mixture (20 µL) contained 10 µL of 2×ChamQ Universal SYBR qPCR Master Mix (Cat. No. Q711-02, Vazyme, Nanjing, China), 0.4 µL of each forward and reverse primer (10 µM), 2 µL of cDNA template, and 7.2 µL of nuclease-free water. *ScGADPH* and *NbActin* were used as the internal reference genes for sugarcane and *N. benthamiana*, respectively. The amplifications were carried out using a LightCycler 96 Real-Time PCR System (F. Hoffmann-La Roche, Basel, Switzerland). The qPCR cycling conditions were as follows: initial denaturation at 95 °C for 30 s, denaturation at 95 °C for 10 s, and annealing and extension at 60 °C for 30 s, followed by a melting curve from 55 °C to 95 °C. Relative transcript expression levels were calculated via the 2^−ΔΔCt^ method using the mean ± SEM. The target-specific primers are listed in Supplementary Table S1.

### 4.4. Subcellular Localization of ScGID1

An enhanced green fluorescent protein (eGFP) report gene was fused to the C-terminus of ScGID1 and cloned into the binary expression vector pCHF3 (*CaMV 35S* promoter) to generate the recombinant plasmid pCHF3-*ScGID1*-*eGFP* using the ClonExpress MultiS One Step Cloning Kit (Cat. No. C113-01, Vazyme, Nanjing, China) following the manufacturer’s instructions. The primers used are listed in Supplementary Table S1. The recombinant plasmid pCHF3-*ScGID1*-*eGFP* and pCHF3-*eGFP* (negative control) were transformed into *Agrobacterium tumefaciens* strain EHA105 via electroporation, respectively. The *A. tumefaciens* cultures containing pCHF3-*ScGID1*-*eGFP* or pCHF3-*eGFP* were resuspended in infiltration buffer [10 mM MES (Cat. No. A420766, Sangon Biotech, Shanghai, China), pH 5.6; 200 μM acetosyringone (Cat. No. A419125, Sangon Biotech, Shanghai, China); 10 mM MgCl_2_ (Cat. No. A100288, Sangon Biotech, China)] to OD600 = 0.6, and then infiltrated into the 4-6-leaf aged *N. benthamiana* using a sterile needle-free syringe as described previously [71]. Fluorescence was observed and imaged under a confocal electron microscope (Leica-TCS-SP8 MP; Leica Microsystems, Deerfield, IL, USA).

### 4.5. Bimolecular Fluorescence Complementation Assays

To explore the interaction between ScGID1 and ScGA20ox2 or ScGAI, the CDS of *ScGID1* was cloned and fused to the N-terminal of a split yellow fluorescent protein (YFP) in the 2YN vector to obtain the 2YN-*ScGID1*, while the *ScGA20ox2* or *ScGAI* was fused to the C-terminal of YFP to obtain the recombinant plasmids 2YC-*ScGA20ox2* or 2YC-*ScGAI*, respectively. The corresponding recombinant plasmids were transformed into *Agrobacterium tumefaciens* strain EHA105 via electroporation. The *A. tumefaciens* cultures containing 2YN-*ScGID1* were mixed in equal volume with those containing 2YC-*ScGA20ox2* or 2YC-*ScGAI*, respectively. The inoculation of *N. benthamiana* and fluorescence observation were as described above. 

### 4.6. Yeast Two-Hybrid and Yeast One-Hybrid Assays

The cDNA library was constructed using a mixture of different tissues of ROC22 cultivar from ProNet Biotech Co., Ltd. (Nanjing, China). The coding sequence (CDS) of *ScGID1* was amplified and cloned into the pGBKT7 vector to generate the pGBKT7-*ScGID1* bait plasmid. The CDS of *ScGA20ox2* and *ScGAI* were cloned into the pGADT7 vector to obtain the prey plasmids pGADT7-*ScGAI* and pGADT7-*ScGA20ox2*, respectively. For ScGID1-interacting host factor screening, the constructed bait and cDNA library were co-transformed into Y2HGold yeast competent cells using the PEG/LiAC method. Transformants were incubated on SD/−Leu/−Trp/−His (TDO) (Cat. No. PM2151, Coolaber, Beijing, China) agar solid medium at 30 °C for 5–7 days. Colonies with a diameter greater than 3 mm were streak-plated to a higher stringency SD/−Leu/−Trp/−His/−Ade/X-α-gal (QDO/X) (QDO: Cat. No. PM2111; X-α-gal: Cat. No. CX11922, Coolaber, Beijing, China) agar medium. The colonies with growth and turning blue on QDO/X were then cultured in TDO liquid medium, and the corresponding cDNA plasmids were extracted and then transformed into *E. coli* DH5α cells. The positive colonies were determined via PCR and corresponding plasmids were extracted and verified via sequencing. To test the interaction between ScGID1 and ScGA20ox2 or ScGAI, each of the prey plasmids was co-transformed with ScGID1 into the Y2HGold yeast cells and cultured on SD/−Leu/−Trp double dropout (DDO) (Cat. No. PM2222, Coolaber, Beijing, China) medium at 30 °C for 2–3 days. Then, 3–5 single colonies were resuspended in 50 μL of sterilized deionized water and a volume of 10 μL of the resuspended cells was dripped onto TDO and QDO/X agar medium at 30 °C for 3–5 days. The setups pGADT7-*T* + pGBKT7-*p53* and pGADT7-*T* + pGBKT7-*Lam* were used as positive and negative controls, respectively. All of the primers used for recombinant plasmid construction are listed in Supplementary Table S1.

To identify the potential regulatory TFs of *ScGID1*, the potential *CorePro* of *ScGID1* was inserted into the pHIS2 vector. Then, the recombinant pHIS2-*CorePro* plasmid was transformed into the yeast strain Y187 to obtain the bait reporter strain. The minimum inhibitory concentration of 3-amino-1,2,4-triazole (3-AT) (Cat. No. CA1311, Coolaber, Beijing, China) was measured on DDO medium. The construction of the cDNA library for Y187 screening is described above. The cDNA library plasmids were transferred into the bait yeast strains containing pHIS2-*CorePro*, and positive colonies were selected on TDO plates supplemented with 30 mM 3-AT. The prey fragments of the positive colonies were identified via Sanger sequencing. The coding regions of *ScS1FA*, *ScPLATZ*, and *ScGDSL* were cloned into the pGADT7 vector as prey plasmids. The prey plasmids were individually transformed into the Y187 bait strain containing pHIS2-*CorePro*. Transformants were cultured on TDO medium plates containing 0, 10, or 30 mM 3-AT for 3 days at 30 °C. The experiments were repeated three times. 

### 4.7. Split Luciferase Assays

The CDS of *ScGID1*, *ScGA20ox2,* and *ScGAI* were inserted into the JW-like-771-LUC-N (nLUC) and JW-like-772-LUC-C (cLUC) vectors, respectively, to obtain the nLUC-*ScGID1*, cLUC-*ScGA20ox2,* and cLUC-*ScGAI* recombinant plasmids. The recombinant plasmids were then transformed into *A. tumefaciens* strain GV3101 via electroporation. The *A. tumefaciens* cultures were resuspended to OD600 = 1.0, and then the cultures containing nLUC-*ScGID1* and cLUC-*ScGA20ox2* or cLUC-*ScGAI* were mixed in equal volumes and co-infiltrated into *N. benthamiana* plants at the 4–6-leaf stage using a sterile needle-free syringe. After 48 h of cultivation in an insect-free chamber at 25 °C under a 16 h light/8 h dark photoperiod, 1 mM D-luciferin (Cat. No. CL6928, Coolaber, Beijing, China) was applied onto the leaves, and images were captured using a Tanon 5200 Multi Chemiluminescent Imaging System (Tanon, Shanghai, China). All of the primers used for recombinant plasmid construction are listed in Supplementary Table S1.

### 4.8. Promoter Cloning and Cis-Acting Regulatory Element Analysis

A DNA fragment of approximately 2000 bp upstream of the ROC22 *ScGID1* CDS was amplified, and potential promoter activity was analyzed using the PlantCARE database (http://bioinformatics.psb.ugent.be/webtools/plantcare/html/ access date: 21 June 2024) to investigate cis-acting regulatory elements. A fragment of 500 bp containing CAAT-box and TATA-box elements was considered as the potential core promoter region (*CorePro*); specific primers were designed using SnapGene software (Version 5.1.2) and synthesized.

### 4.9. Histochemical GUS Staining Assay

To test the transcriptional activity of the potential *ScGID1*-*CorePro*, the *35S* promoter of the binary expression vector pCHF3 was replaced with *CorePro* to obtain pCHF3-*Core*. Subsequently, a β-glucuronidase (GUS) reporter gene was inserted into MCS to obtain the recombinant plasmid pCHF3-*CorePro*-*GUS*. pCHF3-*CorePro* and pCHF3-*35S*-*GUS* were used as negative and positive controls, respectively. Transient expression was performed in *N. benthamiana* as described previously. GUS histochemical staining assays were performed to examine the expression of the *GUS* gene using a GUS staining kit (Cat. No. SL7160, Coolaber, Beijing, China) according to the manufacturer’s instructions. Photographs were taken by using a Canon D90 digital camera after decolorization in 70% ethanol.

### 4.10. Electrophoretic Mobility Shift Assays

The recombinant MBP, MBP–ScS1Fa, MBP–ScGDSL, and MBP–ScPLATZ proteins were purified from *E. coli* strain BL21 (DE3). The CorePro fragments were PCR amplified and used as the probes. EMSA assays were performed as described previously and we made some modifications [72]. Typical binding reactions contained 150 ng *CorePro* probes, 100 ng of poly (dI-dC) (Cat. No. 20148E, Thermo Fisher Scientific, Shanghai, China) plus 5-, 10- or 15 μg of MBP-tag fusion proteins in the binding buffer [25 mM Tris-HCl (pH 7.5) (Cat. No. A100193, Sangon Biotech, Shanghai, China), 5 mM MgCl_2_, 0.2 mM EDTA (Cat. No. A100322, Sangon Biotech, China), 1 mM dithiothreitol (DTT) (Cat. No. A100281, Sangon Biotech, Shanghai, China), and 2.5 mM ATP (Cat. No. P0756S, New England Biolabs, Ipswich, MA, USA)]. The mixtures were incubated at 37 °C for 45 min, separated on 1.5% agarose gel, and visualized using a Tanon 5200 Multi Chemiluminescent Imaging System (Tanon, Shanghai, China).

### 4.11. Dual-Luciferase Reporter Assay

The potential CorePro of *ScGID1* was inserted into the pGreenII0800-LUC vector to drive a firefly luciferase gene. The coding sequences of the potential transcription regulators (ScS1FA, ScPLATZ, and ScGDSL) were amplified and cloned into the binary expression vector pCHF3. The pCHF3 empty vector was used as a negative control. All of the recombinant plasmids were transfected into *A. tumefaciens* strain GV3101 cells via electroporation. *Agrobacterium* cultures carrying effector or reporter plasmids were resuspended in infiltration buffer to an OD600 = 1.0 and then mixed equally and co-infiltrated into *N. benthamiana* leaves at the 4–6 leaf stage. At 2 dpi, the infiltrated leaves were sprayed with 1 mM D-Luciferin (Cat. No. CL6928, Coolaber, Beijing, China) and incubated for 5 min in the dark at room temperature (RT). Firefly luciferase (LUC) activity was observed and imaged using a Tanon 5200 Multi Chemiluminescent Imaging System (Tanon, Shanghai, China).

### 4.12. Constructs and Transformation of N. benthamiana

A 3×Flag tag was fused to the N-terminus of ScGID1 and cloned into a pBWRA (V) HS vector harboring a *CaMV 35S* promoter and transferred into *A. tumefaciens* GV3101. The plasmid containing *3×Flag* was used as a negative control. The transgenic *N. benthamiana* seedlings overexpressing *3×Flag*-*ScGID1* or *3×Flag* were commissioned to be generated by Wuhan BioRun Biotechnology company (Wuhan, China).

## 5. Conclusions

In this work, the sugarcane gibberellin pathway was found to be involved in the response to *Sporisorium scitamineum* infection, and a gibberellin receptor gene *ScGID1* was obtained. ScGID1 had a homology sequence with the hormone-sensitive lipase family; it was located in the nucleus and cytoplasm and with the highest expression level in the leaves. The ScGID1-interacting proteins were screened using Y2H, and ScGAI1a and ScGA20ox2 were proven to interact with ScGID1 in a gibberellin-independent manner. To our knowledge, this is the first report on the interaction of ScGID1 with a GA biosynthetic enzyme (ScGA20ox2) in sugarcane. A total of 50 potential upstream regulatory factors of ScGID1 were screened, and it was found that ScPLATZ and ScS1Fa inhibited the transcription of *ScGID1*, while ScGDSL promoted its expression, and all three genes were differentially expressed in response to sugarcane smut infection. Overexpression of *ScGID1* in transgenic *N. benthamiana* plants could increase plant height and induce an early flowering phenotype. Our findings in this study not only help to shed light on the understanding of the metabolic regulatory network of sugarcane gibberellin but also expand our knowledge of the interaction between sugarcane and pathogens.

## Figures and Tables

**Table 1:** Gene function annotation of the proteins interacting with ScGID1 in sugarcane.

Number	Gene ID	Functional Description
1	Sh_228D18_p000070	Phospholipase A1-II 7
2	Sh_210G02_p000050	Malate dehydrogenase
3	Sh_265O22_p000030	Alcohol dehydrogenase 1
4	Sh_216J02_p000080	E3 ubiquitin-protein ligase RGLG2
5	Sh_254P24_contig-1_p000020	Similar to the Ubiquitin-conjugating enzyme family protein
6	Sh_234J05_p000030	Bisdemethoxycurcumin synthase
7	Sh_218M08_p000070	Dormancy-associated protein 1
8	Sh_222J11	Aconitate hydratase

## Data Availability

All of the data produced in this research are contained in this report and Supplementary Materials.

## Supplementary Materials

The following supporting information can be downloaded at: https://www.mdpi.com/article/10.3390/ijms251910688/s1.
