# Supplementary figures and images for: Functional Characterization of the Gibberellin (GA) Receptor ScGID1 in Sugarcane
Source: Int J Mol Sci. 2024 Oct 4;25(19):10688. doi: 10.3390/ijms251910688 (PMC11477236; doi:10.3390/ijms251910688)

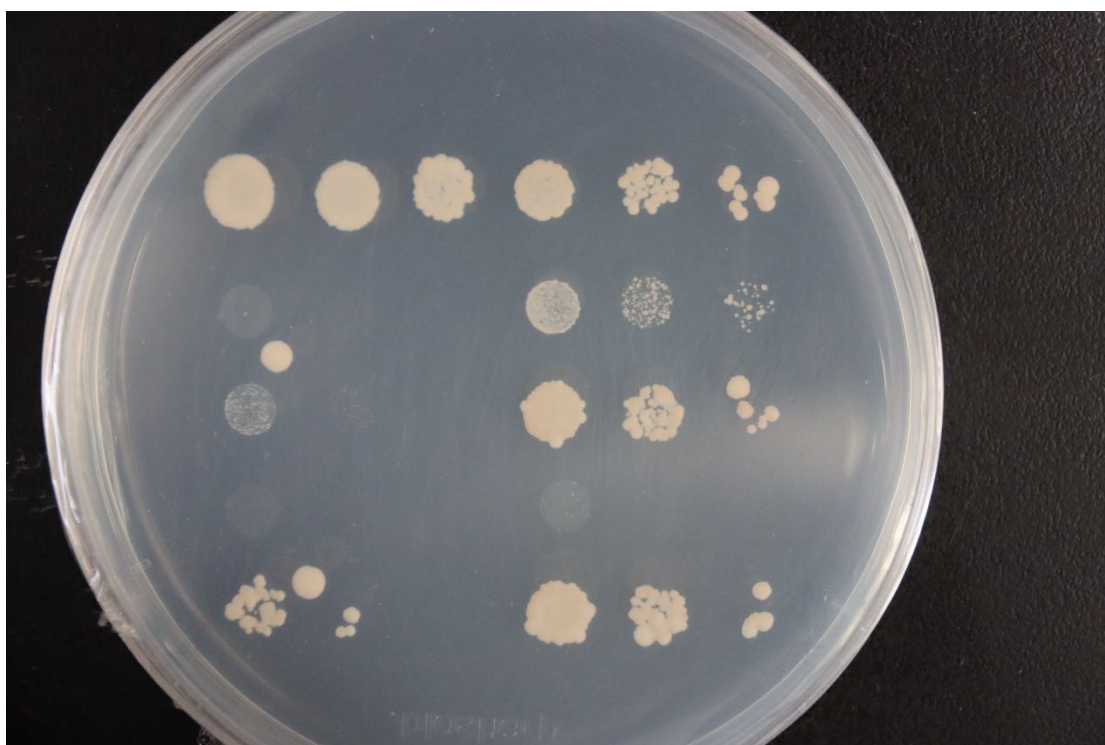

Original image of Figure 4C cultured on TDO.

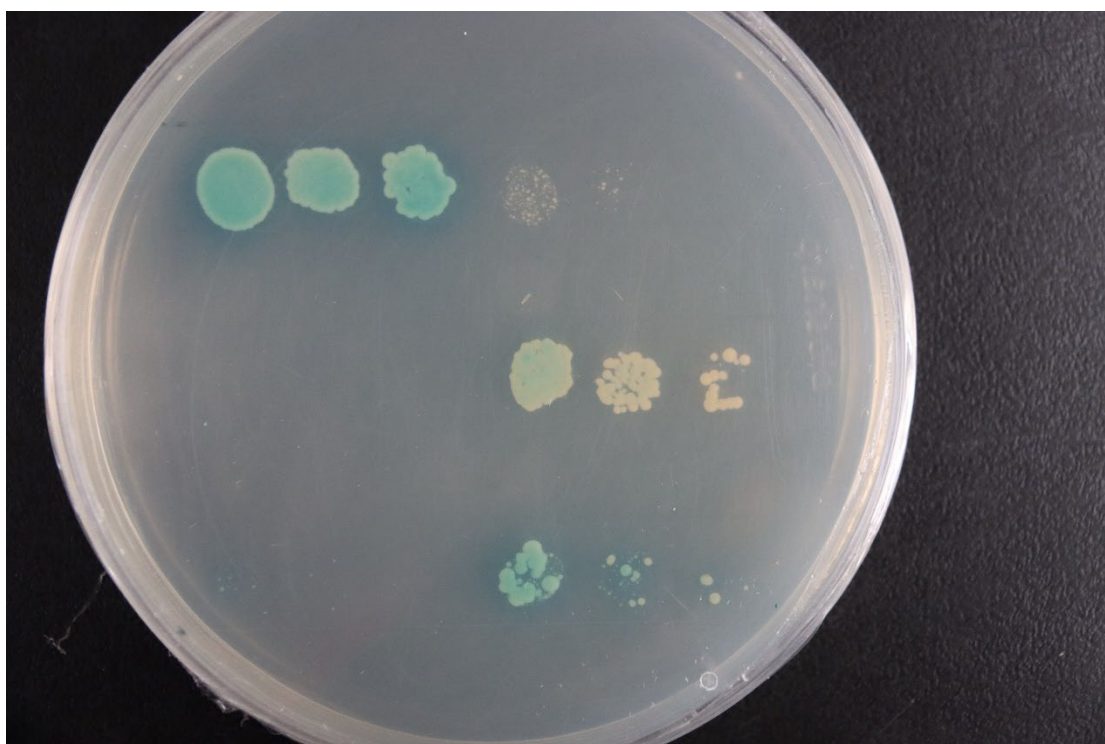

Original image of Figure 4C cultured on QDO/X.

Supplement: Supplementary file 1 [file ijms-25-10688-s001.zip › ijms-3182843-supplementary.pdf]
